# Controllability of protein-protein interaction phosphorylation-based networks: Participation of the hub 14-3-3 protein family

**DOI:** 10.1038/srep26234

**Published:** 2016-05-19

**Authors:** Marina Uhart, Gabriel Flores, Diego M. Bustos

**Affiliations:** 1 Cell Signal Integration Lab, Instituto de Histología y Embriología “Dr. Mario H. Burgos” CCT CONICET Mendoza Facultad de Ciencias Médicas U.N. Cuyo P.O. Box 56 - Mendoza - ZIP 5500 Argentina; 2 Eventioz/Eventbrite Company, Adolfo A Calle 1853, Dorrego, Guaymallén, Mendoza, Argentina

## Abstract

Posttranslational regulation of protein function is an ubiquitous mechanism in eukaryotic cells. Here, we analyzed biological properties of nodes and edges of a human protein-protein interaction phosphorylation-based network, especially of those nodes critical for the network controllability. We found that the minimal number of critical nodes needed to control the whole network is 29%, which is considerably lower compared to other real networks. These critical nodes are more regulated by posttranslational modifications and contain more binding domains to these modifications than other kinds of nodes in the network, suggesting an intra-group fast regulation. Also, when we analyzed the edges characteristics that connect critical and non-critical nodes, we found that the former are enriched in domain-to-eukaryotic linear motif interactions, whereas the later are enriched in domain-domain interactions. Our findings suggest a possible structure for protein-protein interaction networks with a densely interconnected and self-regulated central core, composed of critical nodes with a high participation in the controllability of the full network, and less regulated peripheral nodes. Our study offers a deeper understanding of complex network control and bridges the controllability theorems for complex networks and biological protein-protein interaction phosphorylation-based networked systems.

During the last decade, protein–protein interaction network (PPIN) analysis became essential to study the exponentially grown human protein interaction data. Structural analysis of these networks can lead to new insights into biological systems and is a helpful method to propose new hypotheses^1^,^2^. Network science has offered deep insights into the structure and dynamics of complex networked systems^3^. However, how dynamics of complex networks is controlled is still an unanswered question^4^. According to the control theory, a dynamical system is controllable if, with a suitable choice of inputs, it can be driven from any initial state to any desired final state within finite time^4^,^5^,^6^. To analyze the robustness to control a network under unavoidable failure, Liu classified each node in a network into one of the following three categories: (1) a node is ‘critical’ if in its absence we have to control more driver nodes to control the network, i.e. N’D > ND (ND: driver nodes, set of nodes (driven by different signals) that can offer full control over the network. N’D: set of driver nodes in any new condition different to the initial condition in which ND was calculated). This means that removing one critical node in the middle of a directed path will cause an increase in ND. (2) a node is ‘redundant’ if in its absence we have N’D < ND. For example, removing one redundant leaf node in a star will decrease ND by 1. (3) a node is ordinary if in its absence we have N’D = ND. For example, removing the central hub node in a star will not change ND at all^7^. Most networks have a few critical nodes, being most of them redundant. This means that they have a role in some control configurations but that the network can still be controlled in their absence. The other element in PPINs, the links between proteins (named edges) are usually considered identical, although the physical characteristics of protein-protein interactions are largely determined by their (different) interface structures. In particular, there are big differences between the classical and more studied domain-domain interactions (DDIs) and the domain-intrinsically disordered region (IDR) interactions. The later are segments of a protein that do not fold completely, remaining flexible^8^. The main difference relay on the fact that IDRs generally contain eukaryotic linear motifs (ELMs) within the disordered region component. ELMs are short peptides composed of particular sequence patterns that bind to specific protein domains^9^. DDIs usually display 10^3^–10^6^ folds stronger affinities than domains-linear motif interactions (DLIs). Whereas DDIs tend to be characterized by large, strong interfaces between two globular domains, DLIs are typically composed of small, weak interfaces between a domain and a short peptide. The implications of DLIs and DDIs biological roles were investigated by Kim and coworkers, who found that DLIs are more likely to connect proteins between different biological modules, whereas DDIs tend to connect proteins within the same biological module^8^. Interestingly, it has been reported previously that weak interactions are enriched in between-module connections and are important for the proper function of various complex networks. Signaling and post-translational regulated networks are enriched in interactions mediated by the above mentioned ELMs, suggesting that transient interactions mediating connections between modules may be a common design principle in complex networks. ELMs are very important in the evolution and rewiring of PPINs^10^, because of they are often located within intrinsically disordered regions (IDRs) of proteins^11^,^12^. These regions are fast evolving (divergent) sequences and are usually involved in PPINs^2^,^9^,^13^. Eukaryotic proteomes include a large fraction (~30%) of long regions that are natively disordered, and thus do not adopt a fixed structure^14^. These regions have key physiological roles; for example, they are involved as communicators in many cellular signaling pathways^15^. Particularly in phosphorylation, the central post-translational modification (PTM) in most signaling networks, it is clear an enrichment in disorder-promoting residues surrounding the phosphorylation sites^16^. Protein phosphorylation is ubiquitous and affects an estimate of one third of all proteins within the cell^17^. Also, the addition of a phosphate group is one of the most abundant and well understood protein modification. Together with kinases and phosphatases, the regulatory 14-3-3 proteins are essential components of phosphorylation-mediated signaling^18^. The 14-3-3 protein family members interact with phosphorylated partners (phospho-serine or threonine) in a way that they act as “readers” of this widespread PTM in signaling cascades^19^. Indeed, they interact with their partners through the recognition of phosphorylated linear motifs located within disordered regions^19^,^20^. Recognized as a highly expressed protein family, it is now accepted that it is dynamically regulated by acetylation on a critical lysine residue^21^. Here, we studied a directed PPI-phosphorylation-based network analyzing the biological characteristics of its critical nodes and edges. Finally, we simulated a progressive elimination of one 14-3-3 isoform at a time to assess how the network is affected, trying to emulate for example the inhibition of 14-3-3ζ isoform by the acetylation of its critical residue Lys49.

## Results

### Network compilation and characterization

**Figure 1:**
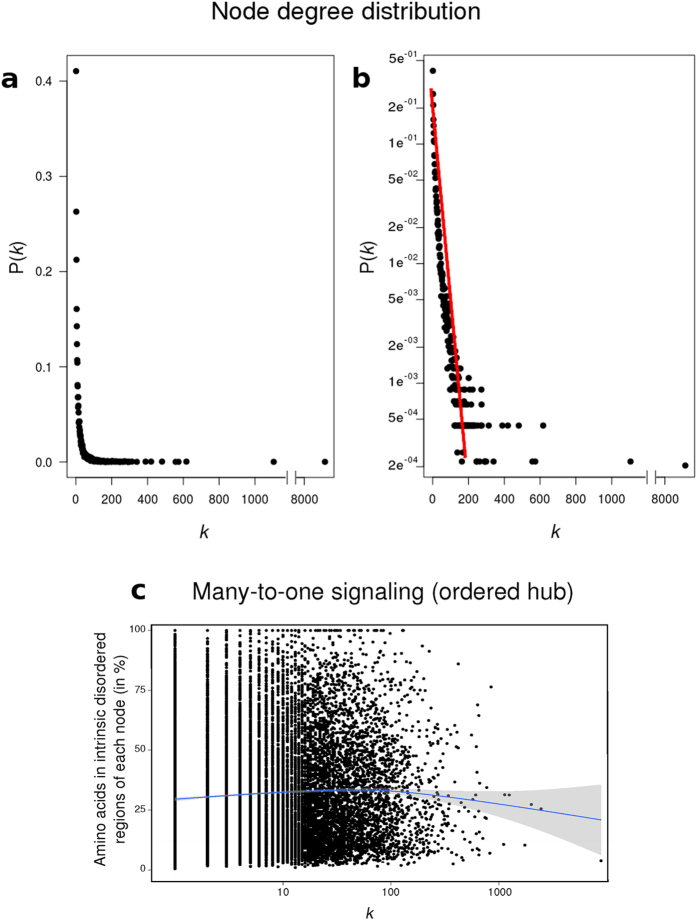
Degree distribution of the network plotted both on a linear (**a**) and logarithmic (**b**) scale. The network follows the power law P(*k*) = A*k*^−3^, which appears as a straight line on a logarithmic plot. Note that every degree between low-degree nodes and hubs appear with a frequency given by P(*k*). (**c**) Percentage of amino acids of each node belonging to disordered regions in relation to the node degree. The percentage of disorder of each node was calculated by the software Cspritz^33^. The non-parametric regression was represented by a blue line.

In order to generate an integrated human phosphorylation-based PPI network, we first collected highly-reliable undirected human PPI data (see materials and methods). The initial integrated human PPIN comprised 264,845 interactions between 16,857 proteins. Then, we filtered the nodes to retain exclusively those empirically reported as phosphoproteins, either in pS/T or pY. This reduced our network to 15,582 nodes connected by 249,102 edges. This undirected network was processed by a state-of-the-art algorithm to convert it into a directed network. To do this, we first excluded of our data (to be transformed in directed) a set of kinase/phosphatase-substrate interactions (KPIs) obtained from^22^, because of these interactions are directed by nature and there is no need to predict them by the Silverbush software. Additionally, we took an unrefined and unfiltered set of 14,427 knockout pairs between 2,870 genes and proteins from^23^ and filtered-out all pairs with *p* ≥ 0.001. Then, these pairs were mapped to their human orthologs. We obtained 83,464 knockout (ko) pairs between 1,943 genes and proteins. KPIs and ko pairs were then integrated with the rest of the PPIs to obtain a physical network of known directed interactions. We removed self loops and parallel interactions. For the latter, whenever both a directed and an undirected edge were present between the same pair of nodes, we maintained exclusively the directed edge. Pairs of edges that were directed in both opposite directions were integrated as a unique undirected edge. The resulting physical network spans 249,102 PPIs of which 83,464 are ko and 49,148 are KPIs. Finally, we applied the Silverbush & Roded’s algorithm^24^ to obtain directions in our undirected PPIN. The resulting network has a typical degree (*k*) distribution (p(*k*) to *k*, where 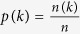, where n is the number of nodes) corresponding to a scale free network (plotted in linear and logarithmic scale, Fig. 1a,b, respectively). We have previously shown the importance of intrinsically disordered regions (IDR) on the increment of *k*^19^,^20^. The Fig. 1c shows the relationship between the percentage of amino acids of each protein (node) in IDR and its *k*. The mean value of disorder is maintained for all values of *k*, meaning that hubs (nodes with *k* in the top 10%) are not particularly intrinsically disordered proteins or proteins with large disordered regions. This shows that in our network, multiple partners potentially gaining very different structures (proteins with IDRs) bind to one -possibly structured- hub in a many-to-one PPI network.

### Characterization of nodes

Our aim was to identify which nodes -or group of nodes- are more important in the control of our network, and by extension to the phosphoproteome. The concept of controllability of complex networks was introduced by Liu and coworkers^4^. We applied their algorithm in order to classify the nodes of our network into one of the three categories defined by them. Critical nodes are the most important in the controllability of the network. The results are presented in Table 1 together with other relevant network properties.

**Figure 2:**
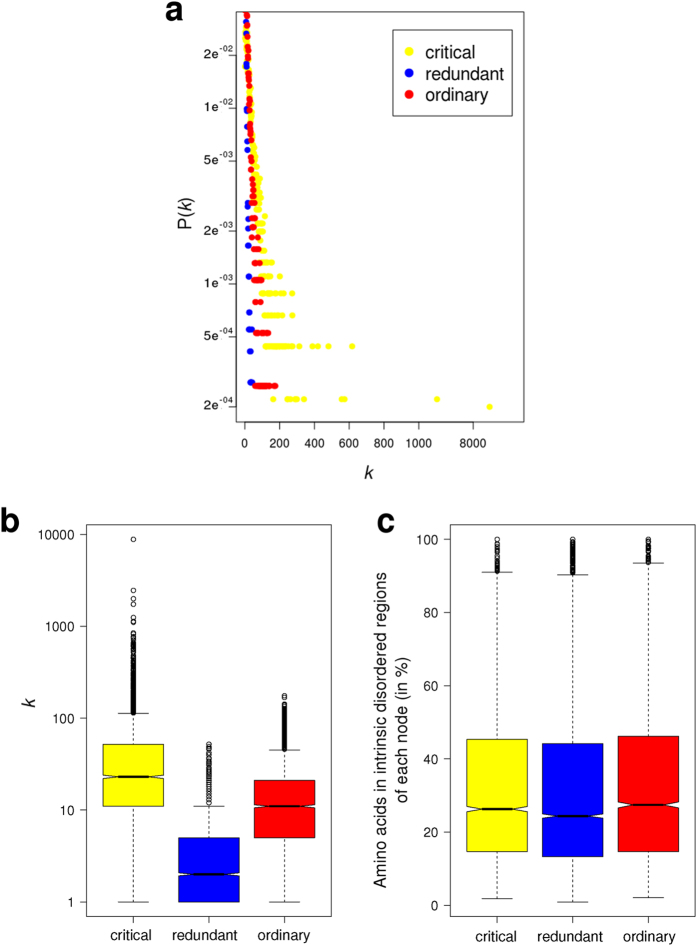
(**a**) Degree distribution of the three node types (critical, redundant and ordinary) plotted on a logarithmic scale. As critical nodes are enriched in high-*k* nodes (hubs), a deviation of the straight line is observed. (**b**) Boxplots of node degree (*k*) for all proteins in the network classified in base on their participation in the network control (critical, redundant or ordinary). (**c**) Boxplots of the content of intrinsic disorder of each protein in the network. The percentage of disordered regions was calculated by the software Cspritz^33^.

**Figure 3:**
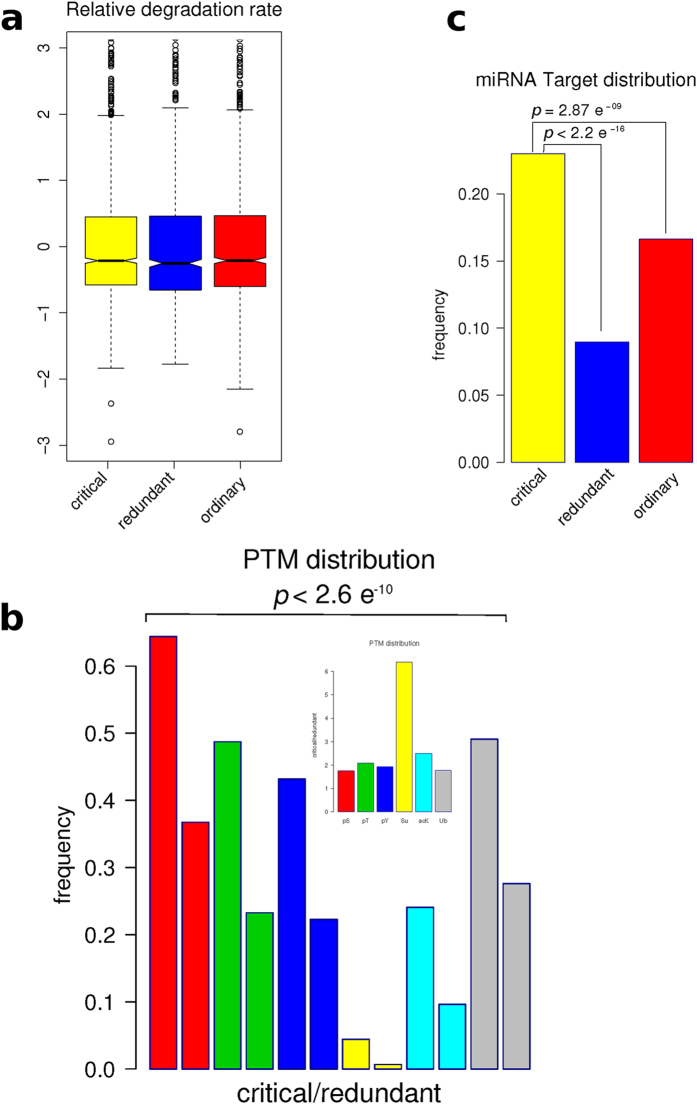
Characterizations of nodes by their kind of regulation. (**a**) Boxplots of the distributions of relative degradation rates for critical, redundant and ordinary nodes. Note that short half-life means high relative degradation rate. (**b**) The bars represent the fractions of PTM regulated sites present in critical/redundant nodes. The inset shows the frequency of each PTM in the critical nodes relative to the same frequency of the PTM in the redundant nodes. (**c**) Nodes of each group as microRNA targets.

**Figure 4:**
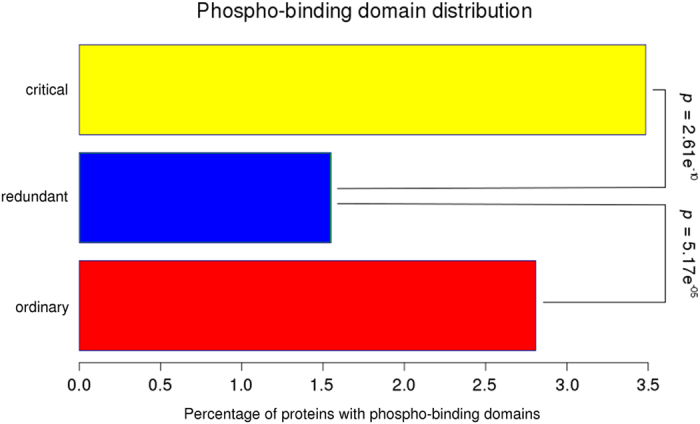
Barplot showing the enrichment of regulatory phospho-protein domains in critical nodes. Statistic values were calculated by Fisher exact test implemented in R.

We characterized the different node types with respect to several common properties. The Fig. 2a shows the interdependence between p(*k*) and *k* for the three types of nodes (critical, redundant and ordinary). Clearly the critical group is enriched in nodes with higher *k*, whereas the redundant group is enriched in those nodes with the lowest *k* (Fig. 2a,b), although the percentage of amino acids inside IDR is maintained statistically invariant between the three groups of nodes (Fig. 2c). To investigate the relationship between the role in the network controllability and protein regulation we characterized each node to get insights about the mechanisms underneath their biological activation or deactivation. In prokaryotic cells, both synthesis and degradation are important processes in the regulation of protein function and enzyme activation. However, this mechanism appears not to have such a high relevance in our human PPIN (Fig. 3a), as no significant differences were observed in the relative degradation rates of the different node types. Other protein modification processes, as post-translational modifications (PTMs) and regulation by miRNAs appear to be statistically more important in the regulation of critical compared with redundant and ordinary nodes (Fig. 3b,c). The Fig. 3b shows that critical nodes are more regulated than redundant nodes by all PTMs analyzed. While phosphorylation, lysine acetylation and ubiquitinilation are twice more present in the critical than the redundant type, sumoylation is more than 6 times present in critical than redundant nodes (Fig. 3b). Another important mechanism of regulation in eukaryotic cells is the inhibition of transduction by miRNAs. We tested if the different node types are targets of miRNAs. We found that this mechanism is also more important in the control of critical nodes compared with the other two types (Fig. 3c). This scenario indicates that critical nodes are target of intense biological regulation. One interesting question is if this regulation is either intra- or inter- group. In the case of miRNA regulation, this is clearly from outside, either for the critical group or the others. However, the question about intra- or inter-group regulation by PTMs had to be tested. We found that phospho-regulatory proteins (those with phospho-binding domains) are prevalent in critical nodes, showing that the regulation of critical nodes by phosphorylation is mainly intra-group (Fig. 4). In other words, critical nodes are regulated by critical nodes when phosphorylation is the mediating PTM.

### Characterization of node interactions

**Figure 5:**
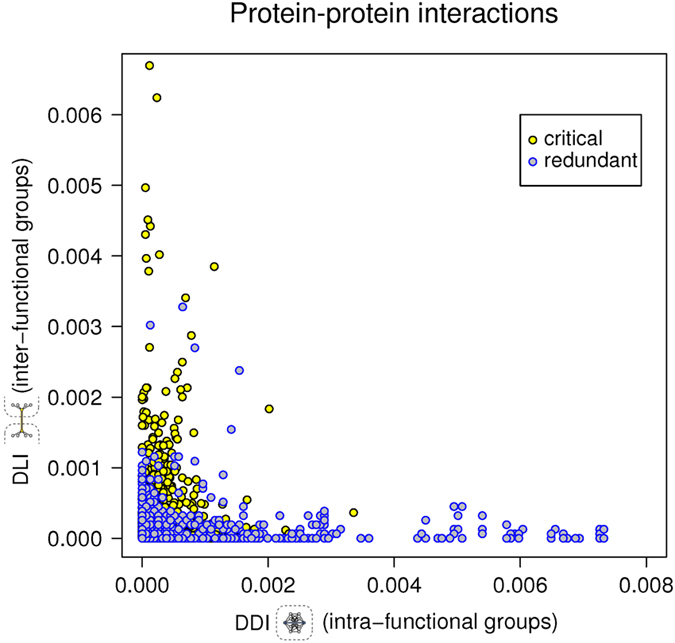
Enrichment of weak domain-to-linear motif interactions (DLIs) and strong domain-to-domain interactions (DDIs) in protein-protein interactions of critical and redundant nodes, respectively. Axis represent the number of interactions divided by total number of interactions and the result divided by the total numbers of proteins in the network (nodes).

**Figure 6:**
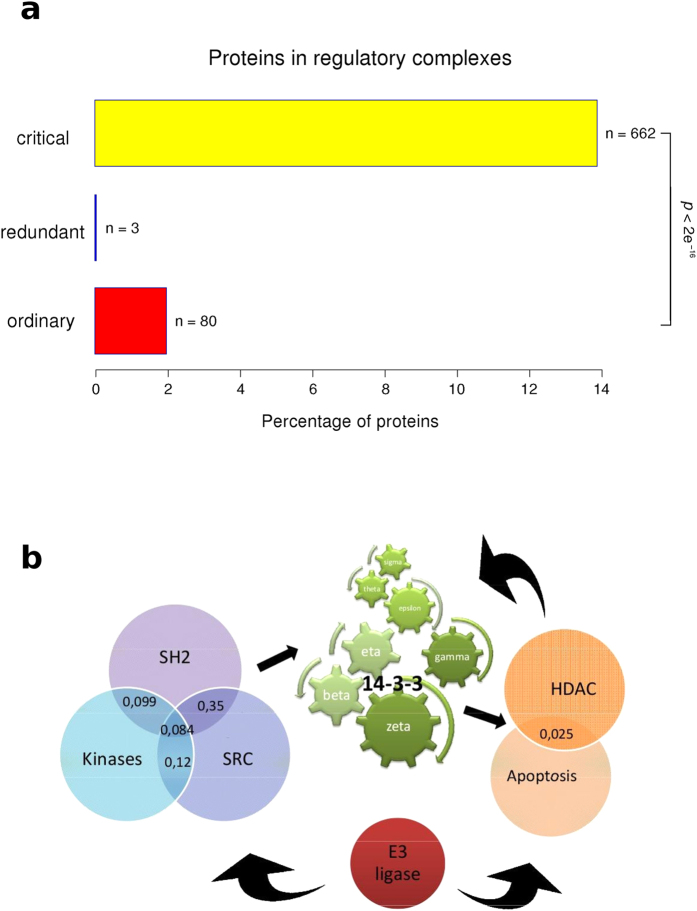
(**a**) Enrichment on stable regulatory protein complexes in critical, redundant and ordinary nodes. Proteins from each group were investigated for their participation in regulatory complexes. Statistic values were calculated by Fisher’s exact test implemented in R. (**b**) Schematic representation of selected regulatory complexes and the 14-3-3 protein family as a common member of these complexes. Numbers in the intersections correspond to the Jaccard index between complexes. This index is calculated by the following formula 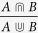 between the components of each complex.

Two kinds of protein-protein interactions are currently accepted, domain-to-domain (DDI) and domain-to-eukaryotic linear motif (DLI). It was postulated that DDIs and DLIs have distinct roles in organizing the modular architecture of human PPI networks. Whereas DDIs tend to link proteins within the same topological cluster or functional group (defined by GO terms), DLIs are more likely to connect different topological clusters or functional groups in the network. To understand how our network is organized, we classified each interaction as DDI or DLI. We found that critical nodes tend to have more DLI interactions whereas redundant nodes have more DDI interactions (Fig. 5). This is also in agreement with critical having higher *k* values than redundant, because ELMs are usually associated with the possibility to accommodate multiple partners in PPIs. The characteristics of critical nodes observed here show this group as very compact and interconnected, in which the edges are mostly weak and transient DLIs, maximizing information transfer at minimal wiring cost (Fig. 5). We then analyzed the enrichment of the functional group “regulatory protein complexes” in critical, redundant and ordinary proteins. We found that this functional group is significantly enriched in critical proteins (Fig. 6a). Although critical proteins interact more through DLIs (that are more likely to connect different functional groups in the network); DLI-critical proteins and critical proteins belonging to the regulatory complexes could be different proteins. Another possibility is that different regions of the same molecule are involved in these differential functions. Liu’s algorithm^4^ classified nodes in each group by the number of in and out edges. The importance of 14-3-3 proteins in the regulation of protein phosphorylation is shown by two facts. The seven 14-3-3 human paralogs are in the top 10 list in Liu’s classification of critical proteins (Table 1), and the 14-3-3 protein family is an essential link between almost all regulatory complexes analyzed. Figure 6b shows a schematic representation of selected regulatory complexes and the 14-3-3 proteins as common members of those complexes.

### Simulation of 14-3-3 paralogs knockdown

**Figure 7:**
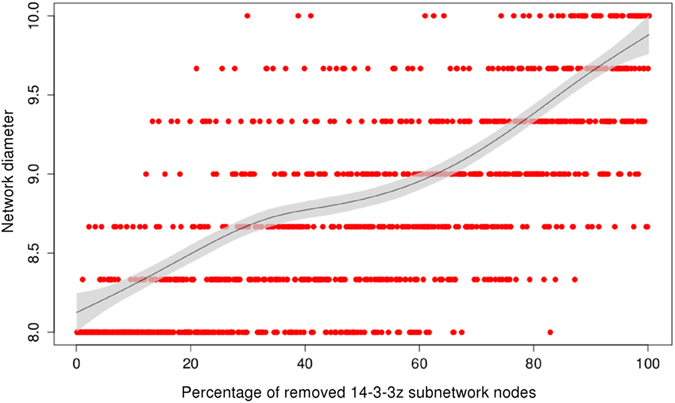


Because of the importance of this protein family and its regulation through a cycle of activation/inactivation by acetylation of specific residues. First, we simulated the knockdown of each 14-3-3 paralog by a complete deletion of nodes of the corresponding paralog in the PPIN and the post-evaluation of the network diameter. The network diameter is proportional to its the interconnectedness (ability of any two nodes to communicate with each other) of a network and it is defined as the average length of the shortest paths between any two nodes in the network. The deletion of all nodes of any 14-3-3 paralog (β, ε, η, ϑ, γ and σ) with the exception of ζ, does not change the original diameter of our network (d = 8). However, the deletion of ζ’s subnetwork produces an increment of 2 units in the network’s diameter (d = 10). In order to know how is the diameter affected by the progressive deletion of 14-3-3ζ, we generated a script to simulate a random partial knockdown of this paralog. This can simulate the biological regulation of 14-3-3 protein family by the acetylation of its essential residue lysine 49, among others. The results are shown in the Fig. 7. The nodes were removed from 0 to 100% in steps of 1% (corresponding from 0 to 6% of the total number of nodes in the full network). This was repeated 3 times and the average diameter was plotted versus the percentage of removed nodes (Fig. 7). The tendency line (black) is biphasic (two-phases). The curve has 2 exponential growth phases (5–30% of removed 14-3-3ζ nodes and 60–90%) separated by a plateau (30–60%), and finishes with another plateau (90–100%).

## Discussion

Systematic studies of human protein-protein interactions are indispensable for deciphering the molecular networks that underlie cellular phenotypes^25^. The cellular responses to signals are frequently initiated by reversible covalent PTMs, especially phosphorylation of already transcribed proteins^26^. These modifications are specifically recognized by interacting proteins, effectively rewiring cellular networks by switching PPIs on or off. Thus, signals are propagated through PTMs that affect conditional PPIs in the cell^18^. In this study, we described the characteristics of a biological protein-protein interaction network based on phosphorylation, one of the most widespread PTM in eukaryotic cells. Network construction is one of the most difficult tasks in order to generate confident results^27^. Our network contains exclusively human proteins with experimentally verified PTMs and protein-protein interactions. Control is a central issue in most complex networked systems, and it is possible to characterize the nodes according to their role in the network control. Until Liu and co-workers^4^ formulated a general theory to explore the controllability in a quantitative fashion, this network’s property was impossible to analyze. They developed an algorithm to identify critical nodes for arbitrary networks, providing a framework in which to address the role of correlations systematically. We applied this algorithm to our directed network. Although there are algorithms to study undirected complex networks, as the exact weights of edges are unknown, at present Liu’s structural controllability framework is the best approach to evaluate the controllability of directed networks^28^.

In order to convert our initially undirected network (it is impossible to obtain directions from protein-protein interaction data) we applied Silverbush’s algorithm^24^. Several strategies have been previously applied to convert undirected to directed PPIs networks^29^, including sophisticated machine learning algorithms^30^. Silverbush’s state-of-the-art computational software was compared to others, and it performed better at conferring directions to a higher percentage of edges in the network. In our previous work^15^, we analyzed the directed network compiled by Vinayagam and colleagues^30^. Our present network includes Vinayagam network and improves it, specially because Vinayagam network was not updated since its compilation in 2011. For the process of transformation of our network in a directed one, we used the same strategy than Silverbush and coworkers did. Kinases and phosphatases with their substrates were considered directed interactions. Also, the information from knowdown gene pairs was used. This reduced significantly the number of edges to transform in directed ones. After the transformation of our network, we applied Liu’s algorithm^4^ to classify each node as critical, redundant or ordinary. Compared with real biological networks as those analyzed in^4^, we found that our network has a small percentage of critical nodes (29%), comparable to metabolic networks from prokaryotic (*Escherichia coli*) and eukaryotic organisms (*Saccharomyces cerevisiae* and *Caenorhabditis elegans*), and to neuronal networks. Given the importance that hubs (defined as the top 10% *k*-nodes) have in maintaining the structural integrity of networks against failures and attacks, in spreading phenomena and in synchronization, we investigated about *k* and other characteristics of this 29% of nodes. We found that this critical group is enriched in regulatory mechanisms, containing at the same time most of the PTMs and the protein domains that regulate those PTMs. However, other mechanisms to regulate the protein/enzyme function/activity as the relative degradation rate (synthesis/degradation rate) are not statistically different in the three groups of proteins. This could be because proteins from the critical group must switch from active to inactive form (and back) very fast. Its degradation, as a regulation mechanism, would be probably inefficient to ensure a fast adaptative response. The diameter of the network is the maximum node eccentricity, and the radius is the minimum. A node is called central if its eccentricity is equal to the radius of the network. Then, the center of the graph is the set of all central nodes^31^. From the results obtained here it is possible to generate a structure of our phosphorylation-based network. It has a central core composed of critical nodes with highers *k* and low eccentricity (Fig. 2b, Table 1) which is highly intra-group regulated, interconnected and it has a important participation in the controllabity of the full network. Peripheral redundant nodes are less regulated with lowers *k* and high eccentricity (Fig. 2b, Table 1). The eccentricity of a node in a biological network can also be interpreted as the easiness of a protein to be functionally reached by all other proteins in the network. Another characteristic that we found of these critical nodes is an enrichment in stable and regulatory complexes, with the participation of the entire 14-3-3 protein family. These protein family members are also at the top-list from the algorithm development by Liu. The common view of 14-3-3s is that they are stable, highly expressed housekeeping proteins. However, it has been shown that 14-3-3s are functionally regulated by acetylation in a particular residue of Lys. This modification disrupts the binding of 14-3-3 to cellular phosphopeptides of RAF1 and KIF1c, two well-know 14-3-3 clients^21^, showing a cross-talk between phosphorylation and acetylation, two of the most important PTMs. We generated a script to simulate this inactivation by acetylation in each paralog of 14-3-3. Only the deletion of the paralog ζ subnetwork, among the seven isoforms of 14-3-3, changed the structure of our network. When we removed one by one each 14-3-3 paralog (except the paralog ζ) the diameter of our network remained unchanged, meaning that the communication between the remaining nodes in the network was unaffected. The removal of these nodes did not alter the path structure of the remaining nodes, and thus had no impact on the overall network topology. This robustness of our network is based in its extremely inhomogeneous connectivity distribution. The deletion of the paralog ζ changed the diameter of our network. Partial removing of this paralog (simulated by random deletion of its edges) changed the diameter (and the topology) of our network. As observed in other studies, directed attacks to hubs produce a monotonic response^3^,^4^,^5^,^32^. The systems must ‘buffer’ the perturbations, which is possible by its redundant wiring. In other words, the system responds tolerating ‘errors’. However, in the case of 14-3-3ζ, the network respond biphasically. Biologically, this could imply that the physiological state of 14-3-3ζ includes a percentage of the protein in an inactive state (probably by acetylation of critical residues like Lys49). Acetylation is a dynamically reversible PTM, and it probably maintains a stable pool of 14-3-3ζ that could be quickly converted to the active form when more active 14-3-3ζ is needed. This hypothesis is at the moment being experimentally tested in our laboratory.

## Methods

### Computer Programming and Statistics

The scripts for data analysis were programmed in Perl and bash (which are freely accessible under request by e-mail to the corresponding author). All statistical analysis to evaluate significance (Wilcoxon rank-sum, Kruskal-Wallis and Fisher’s exact test) were carried out using the R and RStudio statistical analysis package. For the analysis of distributions we used the Wilcoxon rank-sum and Kruskal-Wallis, and for those data without distributions we used the Fisher’s exact test. The similarity between paralog networks was assessed by the Jaccard similarity coefficient (Jaccard index).

### Disorder Predictions

All disorder predictions were made by using the Cspritz^33^ web page (http://protein.bio.unipd.it/cspritz/) as previously done in^15^.

### Integrated human PPI networks

We first collected human PPI data from the following databases: the Human Protein Reference Database (HPRD), release 9^34^; BioGRID, release 3.2.120^35^; IntAct^36^, downloaded 2014; the Database of Interacting Proteins (DIP)^37^, released 2013; Mentha^38^, released 2013 and PINA v2.0^39^. The integrated human PPIN filtered to contain exclusively empirically determined PTMs comprised 249,102 interactions between 15,582 proteins.

### Datasets

PTM datasets (phosphorylation, acetylation, sumoylation and ubiquitinilation) were obtained from the Human Protein Reference Database (HPRD), release 9^34^. Protein containing phospho-regulatory domains were also obtained from the same database. miRNA targets were obtained from^40^. Knockout pair genes were obtained from^23^, and kinase/phosphatase substrate were obtained from^22^. All these datasets were compiled specifically for this paper and are supplied as supplementary information. Relative degradation rates and DDI/DLI datasets were obtained directly from the authors of 8,41, respectively.

### Undirected to directed PPI network conversion

Experimental PPINs are always undirected, and the establishment of edges orientation is a complex problem that has been approached by different strategies. The idea is to maximize the number of pre-specified source-target node pairs that admit a directed path from the source to the target. Here, we used the algorithm proposed by Silverbush and Roded^24^, which tried to maximize the number of pairs that are connected by the shortest (in the original graph) directed path by using a polynomial-size integer linear program formulation. We applied this algorithm to our PPIN. The resulting directed network was compared with the Vinayagan’s network previously used by us in^15^. Both networks overlap in 89% of directed edges.

### Control analysis

We applied the algorithm developed by Liu and coworkers. This algorithm applies the Kalman’s controllability rank condition, where


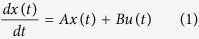


with


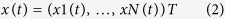


The system described by the first equation is said to be controllable if it can be driven from any initial state to any desired final state in finite time, which is possible if and only if the N X NM controllability matrix


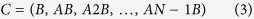


has full rank, that is


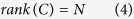


The algorithm identifies the minimum number of driver nodes required to satisfy equation 4.

### Analysis of robustness

Several scripts were generated to randomly invert up to 20% of the directed edges in our network. Then, the set of critical nodes was calculated for each new network condition (with up to 20% of inverted directions) and compared comprehensively to the original set. In the Fig. 1S we plotted the difference (in percentage) in the set of critical nodes between the two conditions as a function of the percentage of randomly inverted edges. Based on this, we are confident that similar results should be obtained if any 20% of the edges were reversed in our directed-PPI network.

### Node deletion simulations

The scripts for node deletion simulation were programmed in Perl. The network diameter was calculated using igraph library implemented in R and Rstudio, and automatically plotted.

## Additional Information

**How to cite this article**: Uhart, M. *et al*. Controllability of protein-protein interaction phosphorylation-based networks: Participation of the hub 14-3-3 protein family. *Sci. Rep.* **6**, 26234; doi: 10.1038/srep26234 (2016).

## Supplementary Material

Supplementary Information

Supplementary Information

Supplementary Information

Supplementary Information

Supplementary Information

Supplementary Information

Supplementary Information

Supplementary Information

Supplementary Information

Supplementary Information

Supplementary Information

Supplementary Information

Supplementary Information

Supplementary Information

Supplementary Information

## Figures and Tables

**Table 1:** Characteristics of the PPIN analyzed in this paper.

**Name**	**Nodes**	**Edges**	**Diameter**	**Eccentricity (median)**
Full Undirected Network	16,857	264,845		
Filtered Undirected Network	15,582	249,102		
Directed Network	15,582	249,102	8	5
Critical nodes	4525 (29%)		*nd*	5
Redundant nodes	7256 (47%)		*nd*	6
Ordinary nodes	3801 (24%)		*nd*	*nd*
**Top ten critical nodes**				
**Node name**	***k***	***k*****_in**	***k*****_out**	
14-3-3γ	565	271	294	
14-3-3β	534	240	294	
14-3-3ϑ	447	63	384	
14-3-3η	299	127	172	
14-3-3ε	506	232	274	
14-3-3ζ	948	393	555	
14-3-3σ	276	123	153	
PAXI1	329	98	231	
ZBT16	146	98	48	
Angiomotin	135	65	70	

*nd* = not determined.
