# Supplementary material for: Controllability of protein-protein interaction phosphorylation-based networks: Participation of the hub 14-3-3 protein family
Source: Sci Rep. 2016 May 19;6:26234. doi: 10.1038/srep26234 (PMC4872533; doi:10.1038/srep26234)
Supplement: Supplementary Information [file srep26234-s15.docx]

Controllability of protein-protein interaction phosphorylation-based networks. Participation of

the hub 14-3-3 protein family.

Marina Uhart^1^ , Gabriel Flores^2^ and Diego M Bustos^1a^

1 Cell Signal Integration Lab, Instituto de Histología y Embriología "Dr. Mario H. Burgos" CCT CONICET Mendoza Facultad de Ciencias Médicas U.N.Cuyo P.O. Box 56 - Mendoza - Argentina. ZIP 5500.

2 Eventioz/Eventbrite Company, Adolfo A Calle 1853, Dorrego, Guaymallén, Mendoza, Argentina.

a corresponding author [dbustos@mendoza-conicet.gob.ar](mailto:dbustos@mendoza-conicet.gob.ar)

Analysis of robustness. Up to 20 % of the total edges directions of the network were randomly inverted. Then, the set of critical nodes was calculated for each new network condition (with up to 20 % of inverted directions) and compared comprehensively to the original set. In the figure 1S we plotted the difference in the set of critical nodes between the two conditions as a function of the percentage of randomly inverted edges.


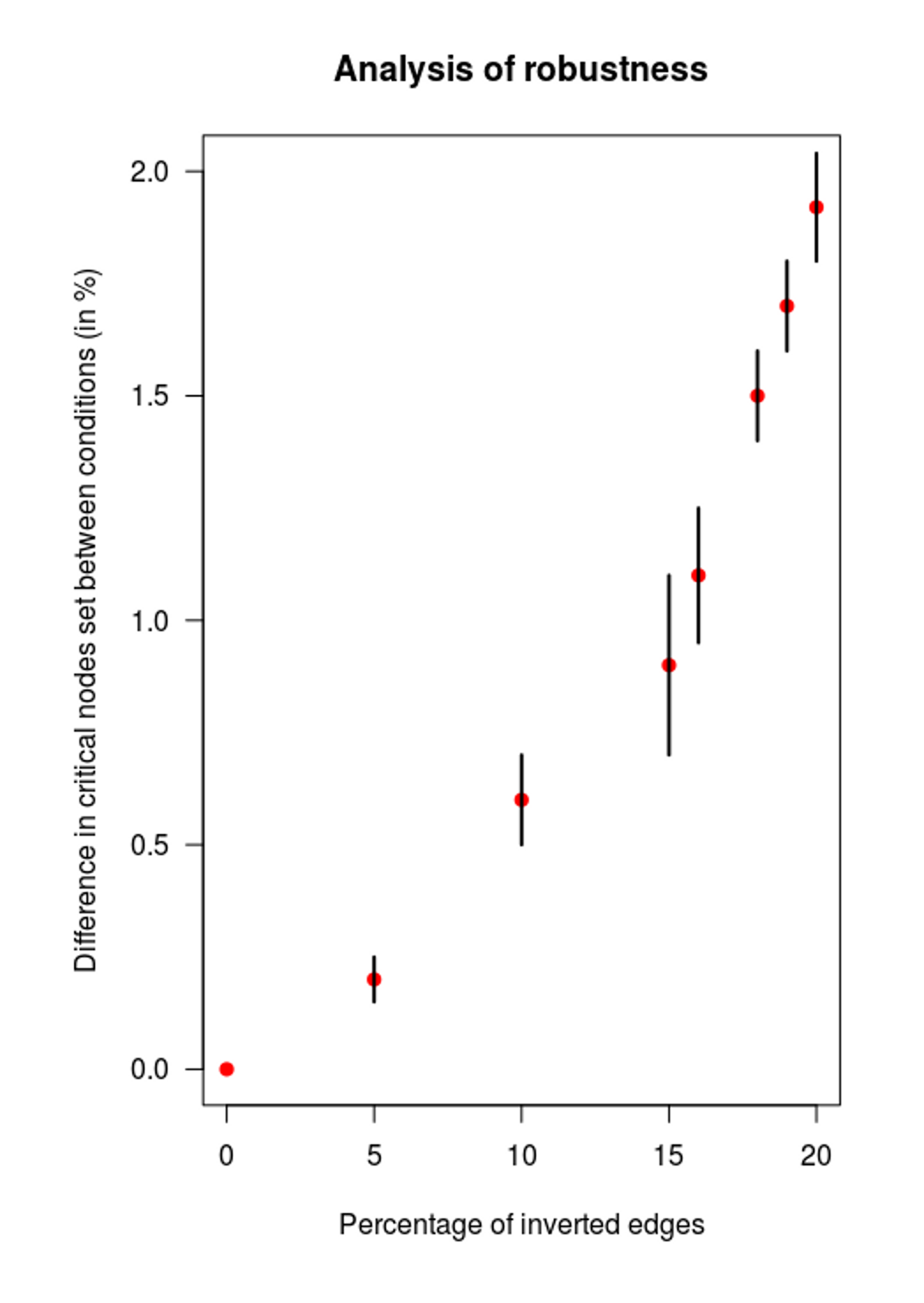


Legend
Figure 1S. Robustness analysis. Plot of the difference in the set of critical nodes after randomly inverting up to 20% of edges directions compared to the set of critical nodes of our original directed network, as a function of the percentage of randomly inverted edges.
